# Evaluation of Somatic Hypermutation Status in Chronic Lymphocytic Leukemia (CLL) in the Era of Next Generation Sequencing

**DOI:** 10.3389/fcell.2020.00357

**Published:** 2020-05-19

**Authors:** Sanjeev Kumar Gupta, David S. Viswanatha, Keyur P. Patel

**Affiliations:** ^1^Laboratory Oncology Unit, Dr. B.R.A IRCH, All India Institute of Medical Sciences, New Delhi, New Delhi, India; ^2^Division of Hematopathology, Mayo Clinic, Rochester, MN, United States; ^3^Department of Hematopathology, The University of Texas MD Anderson Cancer Center, Houston, TX, United States

**Keywords:** CLL, somatic hypermutation, NGS, clonality, immunoglobulin heavy chain

## Abstract

Somatic hypermutation (SHM) status provides an important prognostic indicator for chronic lymphocytic leukemia (CLL), a very common type of mature B-cell leukemia. Owing to the adverse prognosis associated with an unmutated immunoglobulin heavy chain variable (IGHV) status, SHM testing is performed as a standard of care in CLL. Conventionally, SHM testing has been performed using labor intensive and primarily analog Sanger sequencing method following PCR amplification of the clonal immunoglobulin heavy chain gene rearrangements in CLL cells. In comparison, recent availability of next generation sequencing (NGS) allows more versatile detection and direct identification of clonal immunoglobulin gene rearrangements in neoplastic B-cell populations. The ability to identify specific clonal IGHV signature(s) in both baseline (diagnostic) and post-treatment settings enables unique clinical applications of NGS such as determination of SHM status, minimal residual disease (MRD) monitoring, clonal heterogeneity and B cell receptor IG stereotypy. We provide a review of current practices and recommendations for SHM determination using NGS including examples of difficult cases.

## Introduction

### IGHV Rearrangements and Somatic Mutations in CLL

**FIGURE 1:**
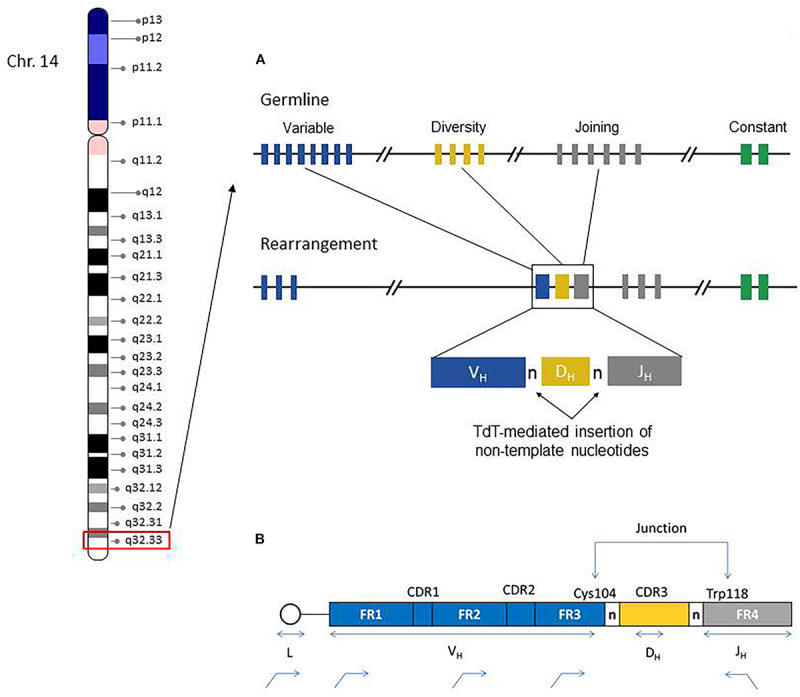
Chromosome 14 ideogram showing genomic location of the IGH gene region (red box). The right side diagram schematically illustrates the germline configuration of V, D, and J IGH genes (top) and the rearranged IGH gene in a given B-cell. Remarkable IGH repertoire diversity is generated by the rearrangement potential of many V, D, and J genes, as well as the effects of junctional nucleotide alterations (trimming and additions of random nucleotide pool bases by the enzyme terminal deoxynucleotidyl transferase, TdT) **(A)**. IGH Rearrangement and Junctional Sequences. Leader primers typically provide a longer sequence as compared to the framework primers. Evaluation of junctional sequences including the Cys104 and Trp118 anchors is essential for SHM determination **(B)**.

The IGH region genes encode the antigen binding variable (V) and isotype-specific constant (C) regions of immunoglobulin (IG) heavy chain proteins and their combinations generate tremendous sequence diversity that is important to effectively identify a variety of antigens (Tonegawa, 1983). The immunoglobulin heavy chain is coded by multiple genes, including the variable (V), diversity (D), and joining (J) genes, which are further organized into four relatively conserved framework regions (FR1, FR2, FR3, FR4) and three variable complementarity determining regions (CDR1, CDR2, CDR3) (Figure 1). The FR1/CDR1, FR2/CDR2 and FR3 regions are encoded by the IGHV gene, whereas the CDR3 is formed from the combination of the 3′ part of the IGHV gene, the IGHD gene and the 5′ part of the IGHJ gene. In addition, a variable number of “non-templated” nucleotides (N) and palindromic (P) nucleotides are added at the V-D and D-J gene junctions, substantially increasing sequence diversity. As such the CDR3 sequence represents a highly unique region of the rearranged IGH gene for any given B-cell.

During B-cell development, there is a random rearrangement of IGHV (*n* = 38–46), IGHD (*n* = 23), and IGHJ (*n* = 6) genes (Figure 1). Only those IG gene rearrangements that are productive and, thus, functional are retained in B-cells and combined with similarly functional rearranged immunoglobulin light chain gene products (kappa or lambda) to form a complete immunoglobulin receptor. The B-cells without productive IGH rearrangements undergo apoptosis.

IGH gene rearrangements in B-cells undergo additional somatic mutations on exposure to antigen as part of the germinal center reaction in lymphoid tissues. These acquired or somatic hypermutations (SHM) are mediated by the enzyme activation induced cytidine deaminase (AICD) and mainly involve nucleotide base changes in the CDR regions. SHM occurs in antigen-activated germinal center B cells and improves the fitness of cells involved during a polyclonal immune response through the process of affinity maturation, which optimizes antigen epitope binding by immunoglobulins (Neuberger and Milstein, 1995; Di Noia and Neuberger, 2007; Pilzecker and Jacobs, 2019). SHM is thus an important aspect of humoral immunity.

As a result of SHM, the nucleotide sequences of IGH gene rearrangements in affected B-cells are different from their corresponding germline counterparts. In the setting of CLL, SHM status is used to define distinct prognostic subgroups. An “unmutated” clonal IGH gene rearrangement is defined as having very high nucleotide sequence identity to its closest germline IGHV gene. “Mutated” CLL in contrast is characterized by IGHV sequence deviating by some percentage relative to its germline reference. By convention, the SHM status in CLL is reported on the dominant clonal population recognized at diagnosis and is defined as a deviation of >2% (mutated status) or ≤2% (unmutated status) from the closest germline IGHV reference sequence. As confirmed by several studies (Damle et al., 1999; Hamblin et al., 1999; Kröber et al., 2002), unmutated CLL is associated with generally poorer outcome, whereas mutated CLL shows less aggressive disease course. Further sub-categorization is becoming apparent with the recognition of specific subsets of CLL based on constrained features of the IGHV CDR3 [see following section on B-cell receptor (BCR) stereotypy]; these subsets are also prognostically significant and may be independent of SHM status. For example, CLL cases having IGHV3-21 rearrangements (particularly those belonging to subset #2) are an exception in that these patients have a worse prognosis regardless of SHM status (Tobin et al., 2002; Thorsélius et al., 2006; Baliakas et al., 2015).

### Somatic Hypermutation Testing Using Sanger Sequencing

Conventionally, SHM analysis in CLL has been performed using Sanger sequencing of the IGHV domain using either DNA or RNA as starting material. This approach, considered gold standard method for determining the SHM status, involves two steps: a PCR and capillary electrophoresis based method to detect clonality, followed by automated fluorescent dye-terminator Sanger sequencing. As indicated the SHM assay evaluates the level of sequence deviation between the CLL IGH gene and the closest matched germline sequence counterpart in order to assign unmutated or mutated status based on the 2% threshold as described above. Web-based IG germline sequence databases such as IMGT (International ImMunoGeneTics Information System^1^) and IgBlast^2^ have greatly facilitated this analytic process. Despite being in use for many years, this assay is still not uniformly performed in many clinical laboratories due to limitations of labor-intensiveness, technical complexity and limited scalability. While this technique generally works well in cases with a single clonal IGH rearrangement, cases with >1 rearrangement, as seen in up to 10% of cases (Langerak et al., 2011), can be extremely difficult to interpret due to the inability to reliably quantify the relative abundance of individual clones. The incidence of cases with >1 rearrangements could also be under-appreciated using Sanger sequencing.

Recent availability of massively parallel sequencing, also known as Next Generation Sequencing (NGS), offers the ability to unambiguously determine the individual clonal sequences and their relative proportions. The ability to determine specific sequences of each of the clones provides a better understanding of the intra-tumor and inter-tumor heterogeneity and clonal evolution. The ability to determine the specific sequence of the clone in the pre-treatment setting allows detection of measurable minimal residual disease (MRD) following therapy. In recent years, many groups have shared their experiences of using NGS assays for SHM testing in CLL (Blachly et al., 2015; McClure et al., 2015; Stamatopoulos B. et al., 2017). Below we summarize significant findings from these studies to provide a better understanding of NGS-based SHM testing in CLL. In addition, we provide considerations for clinical validation and reporting of SHM testing including examples of instructive clinical cases.

## Recent Experience of NGS for Somatic Hypermutations (SHM) in CLL

The use of NGS for SHM analysis was studied by McClure et al. (2015) in which Sanger sequencing was compared to a lab-developed NGS strategy using the Ion Torrent PGM platform (ThermoFisher, Waltham, MA, United States). NGS data was processed with a combination of methods including on-board software and IMGT tools to align reads to germline IGH reference sequences and for SHM percent calculations. Alternate visualization of NGS data was also accomplished using proprietary vended software and the Integrated Genome Viewer (IGV, Broad Institute, Cambridge, MA, United States) to find clonal populations and establish consensus sequences for IMGT alignments. While both methods demonstrated comparable accuracy, distinct advantages of NGS included batch processing (higher efficiency), direct clone determination, cost-competitiveness (with run batching) and the ability to identify >1 dominant clonal IGH rearrangement in some cases. Even though either method requires significant user skill and experience with specific software applications and data analysis, the read pile-ups in NGS data readily generated consensus clonal sequences compared to the more laborious approach of creating clean sequence contigs using Sanger method. Nevertheless, some methodologic inconsistencies are evident in this study, including the use of leader-specific primers for PCR/Sanger sequencing versus family specific FR1 primers for the NGS method. The use of FR1 primers may result in inaccurate IGH SHM calculations because of the need for placement of sequencing primer within the V-domain, leading to a truncated target region for analysis; however, the authors note that this did not substantially impact the mutation status assignment of CLL cases. Despite the greater complexity inherent in NGS methodology, the overall hands-on time of test performance and analysis was found to be comparable. Practically speaking, the semi-automated data analysis of NGS was considered to make the assay interpretation less complex in most instances. The PGM-based method workflow was also noted to be assay agnostic, and so can lead to laboratory work and cost efficiency if samples with different sequencing targets are batch processed simultaneously, in order to maximize utilization of the sequencing chip.

Stamatopoulos B. et al. (2017) employed the LymphoTrack IGH Somatic Hypermutation Assay Kit (Invivoscribe, San Diego, CA, United States) on complementary DNA (cDNA) from purified CD19+CD5+ cells, and found multiple productive IGH clonal rearrangements in almost 25% of CLL patients tested. Since this finding was rarely observed with earlier test methods, it was cross-checked after excluding the contamination by normal B cells and using alternative bioinformatics approaches. The finding of multiple clones may partly be due to higher sensitivity of NGS. The mixture of DNA templates could be amplified and sequenced more reliably with NGS compared with Sanger sequencing. Furthermore, the results could be confirmed when IGHV was amplified using specific VH family leader primers in patients found to have multiple rearrangements by NGS. Significantly, the patients could be categorized based on the IGHV NGS profiles into five subgroups: multiple hypermutated (M) clones; 1 M clone; mix of M and unmutated (UM) clones; 1 UM clone (including V3-21 cases); and multiple UM clones. These subgroups had different disease outcomes depending on the type of clones, as measured by treatment free and overall survival. The use of NGS was found to improve the stratification of CLL and its clinical management, and could help in patient selection for novel therapies. The presence of subclonal heterogeneity in CLL also challenges the current concept of “monoclonality” in malignant lymphoid disease and suggests greater biologic complexity with possible consequences for treatment responses and outcomes in some patients.

Blachly et al. (2015) used next-generation RNA sequencing (RNA-seq) and were able to obtain the complete sequence of IGH transcripts from unselected RNA-seq reads. The multidimensional nature of RNA-seq transcriptome data with it high resolution and high dynamic range was found useful not only for IGHV SHM status determination, but also for simultaneously assessing global gene expression, and identifying known and novel genetic variants. It was concluded that unbiased next-generation RNA-seq can potentially replace the current Sanger sequencing based assay for accurate determination of IGHV sequences, SHM status and additional tumor genetic features.

Wren et al. (2017) have worked on the comprehensive detection of translocations and clonality in lymphoproliferative disorders. They showed that NGS can be utilized to sequence the V-(D)-J gene rearrangements using the EuroClonality-NGS panel based on probe capture methodology. This approach simultaneously assesses clonality, clonal relationships and evolution, IGHV SHM, and can nominate targets for measurable residual disease. More comprehensive NGS panels may be designed to also include recurrent translocations and genetic alterations relevant for diagnosis and prognosis in lymphoproliferative disorders, along with IGHV SHM analysis in a single assay. Relatively unbiased analysis also permits evaluation of the immunoglobulin gene repertoire. One of the goals of deep sequencing of the immune repertoire is to assess the response of the repertoire to its antigenic environment. An overview of statistical and modeling methods was reviewed by Six et al. (2013) to analyze the immunoglobulin and T-cell repertoires in lymphoid proliferations (Six et al., 2013).

### Additional Applications Supported by NGS-Based IGHV SHM Testing

#### MRD Monitoring

Minimal residual disease assessment in CLL can be done using NGS, typically using the same clone identified at diagnosis for SHM determination (Logan et al., 2011). As compared to the currently accepted methods for MRD assessment like allele-specific oligonucleotide PCR (ASO-PCR) and multiparametric flow cytometry (FCM), NGS based method offers unique advantages for MRD analysis. ASO-PCR is highly sensitive but is not commonly performed given laborious technical requirements, including the synthesis and validation of patient clone-specific primers. Although FCM is widely available, immunophenotyping assays often have limited analytical sensitivity. NGS based assays of IGH VDJ segment rearrangements, on the other hand, combine the sensitivity and wide applicability of both allele-specific PCR and FCM assays while using consensus PCR primers and avoiding the need for patient-specific primer generation. A combined approach using multiparameter flow cytometry and high-throughput sequencing for highly sensitive CLL-MRD analysis has also been described by Rawstron et al. (Rawstron et al., 2016).

#### B-Cell Receptor IG Stereotypy

Next generation sequencing based analysis in CLL can also be employed for receptor stereotypy analysis. Receptor stereotypy in CLL is defined as the presence of almost identical BCR immunoglobulin (IG) peptide motifs in unrelated CLL patients. Given the massive diversity of IG rearrangements, the statistical probability of any two CLL patients sharing the same or highly similar clonal IG gene products should be exceedingly small, but surprisingly, one-third of CLL patients can be grouped into stereotyped subsets based on similarities of the IGH CDR3 heavy chain variable region. Receptor stereotypy subset analysis is a further refinement of clinical and prognostic subgroup assessment and improves upon the current binary designation of mutated and unmutated CLL. Recent studies have shown that unrelated CLL patients with near-identical BCR IGH profiles or identical receptor stereotypes are comparable to each other in terms of their clinical characteristics, genetic lesions, epigenetic changes, clinical course, and clinical outcome; for example, the largest stereotyped subset is subset #2 (IGHV3-21/IGLV3-21), which exhibits spliceosome genetic alterations, aggressive clinical course and poor outcome irrespective of SHM status. Thus, receptor stereotypy can be a basis of defining more distinct or homogeneous subgroups beyond SHM analysis. In addition, subsets sharing the same stereotype may potentially be candidates for optimized therapeutic approaches (Tobin et al., 2003; Stamatopoulos et al., 2007; Agathangelidis et al., 2012; Stamatopoulos K. et al., 2017).

## IGHV SHM NGS- Assay Considerations

Relatively unbiased library preparation (i.e., either PCR-generated or capture probe based) and high throughput sequencing of all possible VDJ rearrangements are requirements for accurate assessment of clonality using NGS. These seemingly straightforward goals are, however, challenging to achieve owing to the technical and biologic variability of IGHV rearrangements. Briefly, in comparison to the Sanger sequencing based IGHV SHM analysis, evaluation by NGS is typically performed by amplifying extracted DNA using IGH family specific leader and consensus JH primer PCR master mixes. PCR products are purified using bead-based methods, and the resulting libraries normalized and then pooled for sequencing. Key considerations for NGS-based SHM testing are summarized below.

### Primers

As per the ERIC guidelines, (Ghia et al., 2007; Rosenquist et al., 2017) leader primers should be used so that the whole IGHV region sequence can be obtained (Figure 1) and thus the percentage identity to the closest germline reference gene can be most precisely calculated (i.e., aligned number of target/reference nucleotides X 100) (Sahota et al., 1996; Fais et al., 1998). The use of framework region 1 (FR1)-primers is also employed in diagnostic laboratories in the event that leader primers cannot identify a dominant clonal population. In general, it is recommended that a strategy utilizing leader and FR1 primers be adopted in cases where one of the approaches fails to produce informative sequences. Nevertheless, some additional caveats require consideration when using FR1 family primers. In contrast to PCR templates produced by leader primers for example, the complete IGHV region cannot be assessed with FR1 primers as ∼60 nucleotide bases upstream of the FR1 primer sites are not sequenced; thus the germline identity percentage using FR1 anchored primers is a close approximation compared with leader PCR results (Marks et al., 1991; Kuppers et al., 1993). These small discordances between FR1 and leader primer results might affect the final mutation% calculation. Because FR1 PCR products produce a smaller denominator of nucleotide bases (i.e., IGHV region length) this may lead to slight overestimation of the mutation percentage, which may be relevant near the 2% conventional SHM threshold; however, these differences were generally found to be inconsequential for establishing SHM status and did not typically affect the final clinical results (mutated vs. unmutated) as noted by McClure et al. (2015). This study also identified another potential issue for precise IGHV-gene assignment when using FR1 primers. Because FR1 family specific primers incorporate into all gene rearrangements of a corresponding VH family, any minor segmental sequence differences (i.e., allelic polymorphisms) occurring within the primer region will not be discerned, leading to an equivocal result for the closest germline identity. This is observed when several closely related V-gene alleles are identified but cannot be further delineated (e.g., IGHV4-34^∗^01 or V4-34^∗^04). Practically, this situation does not significantly affect the accurate provision of SHM status in most cases, but requires consideration when interpreting results of FR1 PCR. Conversely, SHM poses a potentially significant technical problem for target enrichment in B-cell IGH analysis. Highly mutated regions in IGH genes may not be recognized during the PCR amplification step due to inefficient consensus primer binding; such clones are thus not represented for analysis.

In summary, for a well-designed assay it is important to include a relatively comprehensive strategy employing either multiple primer sets binding to different locations for each V or J segment; longer primer designs with anchor points at highly conserved positions; or use of full length cDNA with primers positioned outside the V and J regions, which are less likely to undergo SHM (Robins, 2013). It is important to select leader primers for all 7 VH families rather than relying on FR1 only, so that no targets are missed. Similarly, it is better if possible to use two reverse primers (CH and JH) rather than only one, to ensure less false negative PCR results.

### Read Length

As stated, in SHM assays performed by NGS, it is important to perform analysis on full-length reads so that a true representation of each sequence is analyzed. The assay needs unambiguous reads of around 350 base pair (bp) length containing the entire IGHV and junction region, which may be obtained in most cases, although samples with suboptimal DNA quality or degradation may result in failure to achieve optimal read lengths. The commercial LymphoTrack assay (Invivoscribe Inc., San Diego, CA, United States) with leader primers yields paired end sequence reads of approximately 300 bp length each (301 × 2 reads + 8 bases for leader primer = 610 bp) on the Illumina Miseq platform, so that the entire IGHV region of a given rearrangement can be sequenced in a single fragment. The final sequence is also bioinformatically evaluated for features of a productive rearrangement (i.e., absence of stop codon in predicted RNA transcript, in-frame sequence, and presence of the conserved Cys104 and Trp118 residues at the respective junction ends). In essence, complete in frame reads from the primer region across to the junctional Trp118 would be considered productive. McClure et al., reported an average of 350 bp read length using Ion Torrent PGM platform and Ion 400-bp kit using FR1 PCR primers (McClure et al., 2015). The analysis of very short reads is not desirable for SHM analysis and sequences less than 150 bp in length or without the CDR3 sequence are regarded inadequate for assessment.

### Sequencing Platform

The NGS platform selection is based on various factors like desired turnaround time, nature and volumes of samples, complexity of genetic variants to be tested, pre-existing platform in the lab and the ability to achieve a relatively simple workflow in the clinical laboratory, to name a few. Sequencing platforms that support longer reads and have lowest possible error rate will be preferred as they will provide an accurate assessment of SHM. Platforms with higher error rate in certain genomic context and shorter read length are likely to yield a false elevation in SHM%. Platforms with higher AQ score cut-offs (AQ30 or better) for sequencing quality are likely to provide more accurate sequencing data. The rate of true and reproducible mutations over background sequencing error-rate needs to be carefully validated. Currently, the most popular platforms that can achieve longer read lengths using “short read” methods include the Illumina MiSeq and Ion Torrent instruments. In general, Illumina sequencers can achieve sufficient bidirectional reads (2 × 300 bp) with a lower sequence error rate compared with Ion Torrent, but both are acceptable for use with appropriate validation. The advent of long read technology approaches (e.g., Pacific Biosciences, Oxford Nanopore) may provide alternative means of interrogating the full length of IGH rearrangements, but these systems are currently not optimally suited for clinical use or have unacceptably high raw sequencing error rates.

### Controls

**FIGURE 2:**
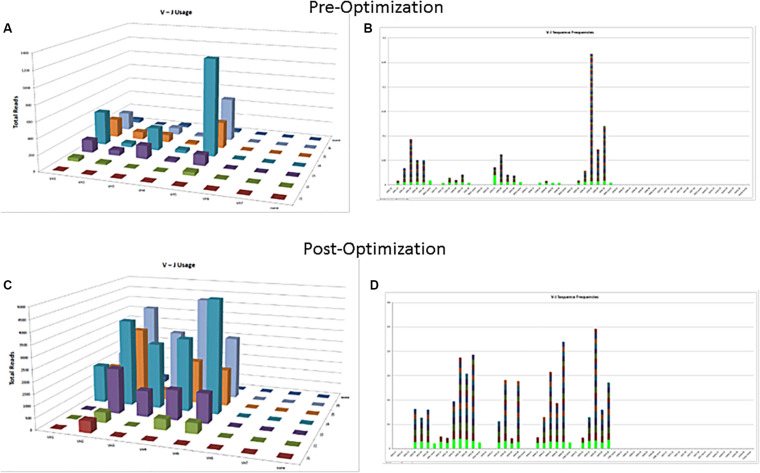
Polyclonal controls showing amplification of many, but not all, V-J combinations. Fewer VJ combinations are detected in a control sample **(A,B)** as compared to an optimized control sample containing mixture of several polyclonal samples **(C,D)**.

The commercially available positive (mutated monoclonal) and negative (polyclonal) controls supplied with the assay kit such as Lymphotrack should always be run with every batch. The polyclonal controls will help in checking the performance of all the primers used in the assay. In case all the families are not being represented in the commercially available polyclonal control, in-house polyclonal samples may be pooled and validated for use, to have a better V region family representation (Figure 2). The initial validation of the NGS assay for SHM can be done using the archived CLL samples with known hypermutation status previously analyzed using the gold standard Sanger sequencing. The controls should be selected to represent a broad range of tumor content and sample types [peripheral blood (PB), bone marrow (BM), etc.] and sample quantity. Positive controls with clones having different VDJ recombination sequences may also be pooled to serve as controls for various primers and could also be prepared to consistently return a specified clonal threshold level (e.g., 10% of total reads). Documentation of family representation is important in ensuring primer performance.

### Sample- and Analyte-Specific Issues

#### Sample Type

Fresh [e.g., fine needle aspirate (FNA), PB, BM, fresh frozen] vs. fixed [formalin fixed paraffin embedded (FFPE), smears] specimens: Currently, commercial IGHV SHM assays used as lab developed tests (LDT) are validated for fresh sample types including PB and BM only, and provided the CLL population constitutes at least 10% of the sample. The minimum tumor criteria are essential to avoid any false negative result. Use of FNA or fresh frozen samples is feasible, but requires additional validation. Use of the assay for SHM analysis is currently not recommended for FFPE samples due to degradation or cross-linking of DNA.

#### Analyte Type: DNA vs. RNA (cDNA)

Current NGS based SHM commercial assays are most often validated for genomic DNA (gDNA) use in majority of clinical labs. A total of 500 ng of input gDNA input is required. Stamatopoulos B. et al. (2017) described results using RNA (reverse-transcribed to complementary DNA, cDNA) as well as gDNA. Per the European Research Initiative on CLL (ERIC) guidelines for Sanger based SHM analysis, both genomic DNA (gDNA) and cDNA, are considered suitable for IGHV analysis (Ghia et al., 2007). The use of RNA has the advantage of preferential identification of functional rearrangements, compared to gDNA, in which non-productive IGH gene rearrangements will also be detected and may need additional analytic evaluation. In contrast gDNA analysis is technically easier to use and allows for better specimen stability (e.g., if sample transportation time is prolonged). In one study, purification of cells had minimal effect on subclonal detection, but cDNA had better sensitivity than gDNA leading to detection of additional clones (Stamatopoulos B. et al., 2017).

### Informatics

#### Read Alignment

The degree of SHM is frequently quantified by comparing the resulting IGHV sequence with the closest consensus germline sequence in the IMGT (International ImMunoGeneTics information system)/V-QUEST (V-QUEry and STandardization) database (see text “footnote 1”). In addition, IMGT provides advanced tools for identifying potential insertion/deletion events in the target sequence. An accurate and detailed sequence analysis of V domains (using IMGT/V-QUEST) and CDR3 regions (IMGT/JunctionAnalysis) can be performed using the online tools developed by IMGT (Yousfi Monod et al., 2004; Brochet et al., 2008; Lefranc et al., 2015). IMGT/HighV-QUEST is a tool for analysis of very large numbers of IG sequences obtained by high throughput NGS deep sequencing assays (Li et al., 2013; Alamyar et al., 2012a, b). IMGT/HighV-QUEST algorithms are similar to IMGT/V-QUEST along with integrated IMGT/JunctionAnalysis. IMGT/HighV-QUEST can analyze around 150,000 IGV domain sequences per batch and perform statistical analysis on around 450,000 sequences. The output from IMGT/V-QUEST consists of 11 compressed files having information about V, D, J gene arrangements, new alleles, detailed junction analysis somatic mutations, and additional data.

Alternatively, the IG sequence analysis tool IgBLAST (see text “footnote 2”) can be used to view matches to the germline V, D, J genes, junction details, IGV framework and complementarity determining regions. IgBLAST can be used to analyze both nucleotide and protein sequences. It can process sequences in batches. IgBLAST can also be used to do simultaneous searches against the germline and local IGH databases to reduce the chance of missing germline V gene matches (Ye et al., 2013). Despite returning identical or highly similar results, each database and its associated bioinformatics toolsets are different and on occasion may result in slight discordances when analyzing a given IGHV sequence. Users should develop an understanding of the possible variations when utilizing these reference databases, or consider cross-database analysis when appropriate.

Vidjil is an open source platform for the analysis of high-throughput V(D)J immune repertoire sequencing data (Duez et al., 2017). The algorithm is intended to work on amplicon-based sequencing as well as hybrid capture-based sequencing. The web application and source code are available for download at http://www.vidjil.org/. Antigen Receptor Galaxy (ARGalaxy^3^) is another open-source web-based tool having capabilities to analyze and visualize the high throughput data of immune repertoire including rearranged V(D)J genes, SHM, and class switch recombination (IJspeert et al., 2017).

Schaller et al., (Schaller et al., 2015) developed a software framework ImmunExplorer (IMEX) to analyze IG and T-cell receptor (TCR) diversity and presence of clonality based on processed NGS data output from IMGT/HighV-QUEST. It helps in visualization as well as detailed statistical analysis of data to identify the unique clonotypes in a sample taking into account the primer efficiency and diversity. IMEX has capabilities of clonotype tracking, diversity analysis and sample comparisons. IMEX is freely available at http://bioinformatics.fh-hagenberg.at/immunexplorer/.

Another web-based, interactive application “ARResT/Interrogate” has been made freely available at http://bat.infspire.org/arrest/interrogate/ by Bystry et al. (Bystry et al., 2017). This application combines various functionalities required for comprehensive *in silico* immunoprofiling. ARResT/Interrogate performs four sequential functions. These include input processing, data selection and filtering, comparative calculations and detailed visualization. ARResT/Interrogate is primarily based on R and Shiny (Shiny is an R package that makes it easy to build interactive web apps straight from R). The analytical core uses “data.table” R package for efficient data handling and can maintain sufficient responsiveness with data generated from thousands of clonotypes and millions of reads. Another favorable aspect of the expanded ARResT toolkit is the ability to upload IGH sequences to query a database (ARResT/AssignSubsets) for common stereotyped BCR IG subsets of prognostic significance.

#### Rollup of Related Sequences

After massively parallel sequencing, the output is analyzed by the bioinformatics pipeline. The paired-end read data are combined, demultiplexed, and adaptor trimmed to generate FASTA and FASTQ files. Primer sequences are trimmed, and exactly similar sequences (duplicates) are removed. Each unique sequence is then aligned using either the commercially provided software, IMGT/V-QUEST or IgBLAST. The output from Lymphotrack for example is grouped based on different IGHV-J combinations, and the final data is loaded into MySQL database. Nearly identical sequences differing by ≤2 bases are merged to accommodate for PCR or sequencing errors. The top 10 merged read sequences are then returned for further analysis, but access to all sequencing data is maintained if required.

#### Calculation of Somatic Hypermutation

The entire IGHV sequence including the CDR3 is compared with the closest germline reference sequence for calculation. The sequence analyzed must include the conserved Cys 104 and Trp 118 residues, and is checked to ensure that the reading frame is preserved (i.e., productive) and that no stop codons are encountered. The percentage identity is calculated using a ratio between the total of IGHV region mutations in the IGHV-D-J rearranged sequence, and the length of the most homologous germline IGHV gene:

$$\begin{array}{c} \mathrm{IGHV}\mathrm{Identity}\left(\%\right)=100-\left\{\mathrm{mutations}\times 100/\mathrm{aligned}\,\mathrm{IGHV}\,\mathrm{region}\,\mathrm{length}\right\} \end{array}$$

The 98% cutoff to differentiate the cases into mutated and unmutated groups is arbitrary and was originally selected from earlier CLL studies to exclude the possibility of polymorphic variant sequences. It was chosen by convention to avoid the need to analyze the corresponding native germline sequence in each patient. Although other cutoff values (e.g., 97% or 95%) for homology determination have also been suggested, it is recommended to follow the 98% cut off for the sake of data uniformity and reporting (Kröber et al., 2002; Lin et al., 2002). Recently, it was also suggested that the absolute percent deviation of IGHV mutation measured as a continuous variable, rather than the static 98% cut-off, predicted survival of CLL patients treated with rituximab, fludarabine, cyclophosphamide therapy (Jain et al., 2018). Thus, it is preferable to report the actual percentage of IGHV homology, in addition to the standard definition of “mutated” or “unmutated” SHM status.

### Result Interpretation and Reporting

**FIGURE 3:**
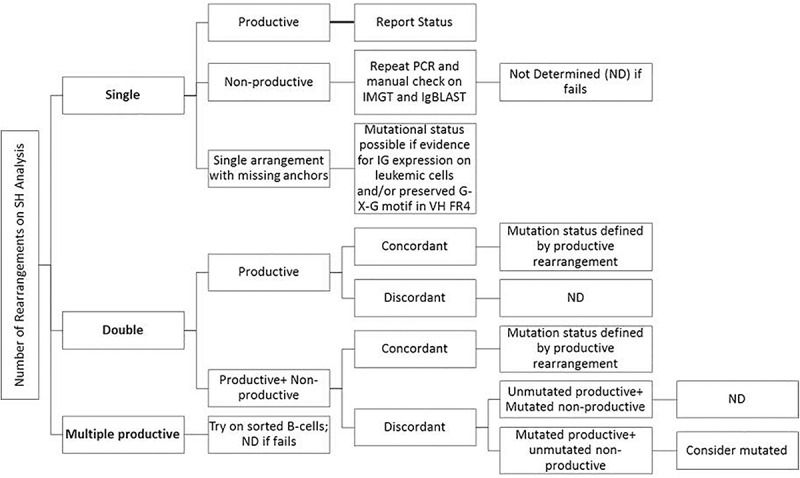
Suggestions for reporting the IGHV gene mutational status in CLL for various types of problematic cases per ERIC recommendations (Langerak et al., 2011; Rosenquist et al., 2017).

Consensus recommendations for SHM were initially provided by ERIC (European Research Initiative on CLL^4^) in 2007 and then later updated in 2011 and 2017 (Ghia et al., 2007; Langerak et al., 2011; Rosenquist et al., 2017). These guidelines, including the minimum reporting elements, were derived for Sanger sequencing methods, but are similarly applicable to NGS-based testing, even though NGS was not specifically mentioned. Specifically, the category “Difficult Cases” that account for up to 13% of cases can be seen at a higher frequency due to the more sensitive and specific nature of NGS as compared to Sanger sequencing. Figure 3 provides an easy-to-use algorithmic representation of ERIC recommendations. The sequence is said to be non-informative or non-productive if there is a stop codon in predicted RNA transcript, the change is not in-frame, or there is absence of the conserved Cys104 and Trp118 amino acids at the junction. Very short reads like sequences less than 150 base pairs long or without authentic CDR3 sequence are also regarded as inadequate for analysis. In routine clinical molecular diagnostic practice, the foregoing informatics analytic considerations can now be managed with vendor-supplied solutions, such as the LymphoTrack software (Invivoscribe). In this application, the first 200 most abundant unique sequences are displayed and used to calculate the overall percentage of the dominant clone, and a table of the top 10 merged sequence reads is also provided. Various clonotypes are also noted (i.e., the sum of all unique sequences with exactly the same VDJ rearrangement but with different length or mutation percentage). Clones with an abundance of <2.5% of total reads may be safely ignored as background normal B-cell contamination, particularly if B-cell enrichment processing with >98% purity has been used pre-analytically. Lymphotrack software incorporates IgBLAST analysis, but many labs also elect to perform analysis with IMGT database to calculate the mutation percentage compared with germline reference, as per the ERIC recommendations.

## Example Cases

The following three cases highlight the application of NGS for IGH SHM analysis.

### Case 1: Two Productive and Concordant Rearrangements

**FIGURE 4:**
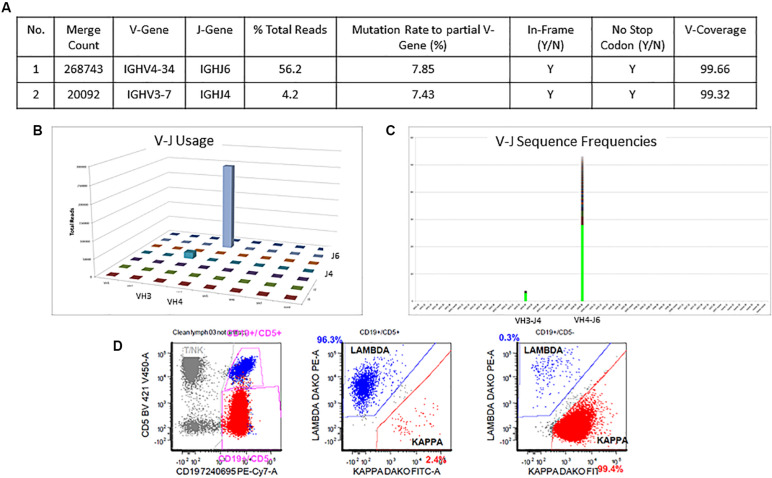
Two concordant productive IGH rearrangements with distinct clonal populations. The major clone utilizing IGHV4-34 and IGHJ6 sequences represent 56.2% of total sequences, whereas the minor clone utilizing IGHV3-7 and IGHJ4 represent 4.2% of total sequences **(A–C)**. Both rearrangements show similar mutation rate (7.8% and 7.4%) relative to the reference V-gene sequence **(A)**. Flow cytometric immunophenotyping shows distinct populations of clonal B-cells with the major subset (red) showing kappa restriction and the minor subset (blue) showing lambda restriction **(D)**.

The detailed Lymphotrack output for case 1 can be seen in Figures 4A–C. The total read count is 477734 against the minimum reportable read count of 180,000. There are two productive clones in this case as both have only in-frame mutations without any stop codon generation. The dominant clone constitutes 56% of total reads, having V4-34 and J6 usage and 7.9% mutation rate. The smaller clone- represents 4% of total reads, with V3-7 and J4 usage and 7.4% mutation rate. Both clones have productive rearrangements and concordant mutational status, i.e., both clones are mutated. The final interpretation for this case is: mutated IGHV status. Such cases represent 0.7% of CLL cases per ERIC. Interestingly, both these clones can be clearly identified separately on flow cytometry (Figure 4D) due to the difference in their light chains. The dominant clone has dimmer co-expression of CD5 with CD19 and is kappa-chain restricted, whereas the smaller clone shows bright co-expression of CD5 and CD19 and is lambda-chain restricted. This is an example showing the correlation of flow cytometry findings with the IGHV gene rearrangements in better understanding of the disease process and its clonal architecture.

### Case 2: Single Non-productive Rearrangement

**FIGURE 5:**
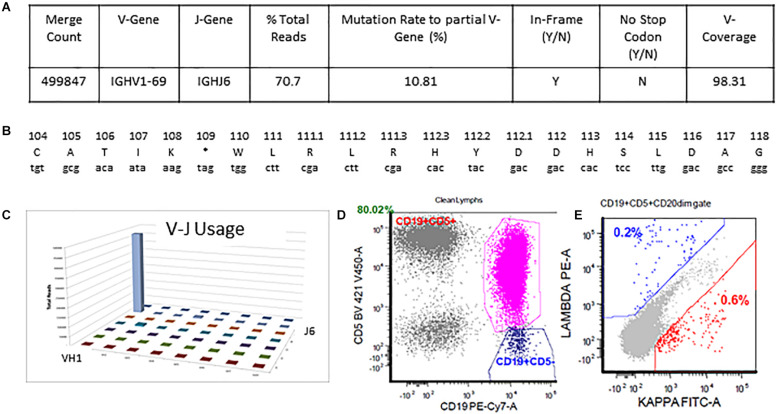
Single non-productive IGH rearrangement in a case with absent surface light-chain expression by flow cytometry. The clonal rearrangement utilizing IGHV1-69 and IGHJ6 sequences represent 70.7% of total sequences and shows a mutation rate of 10.8% relative to the reference V-gene sequence **(A,C)**. Translation of the junctional sequences show a predicted stop codon at position 109 **(A,B)**. Flow cytometric immunophenotyping shows distinct populations of CD5-positive clonal B-cells with absent surface light chain expression **(D,E)**.

The detailed output for case 2 can be seen in Figure 5. The total read count is 706770 against the minimum reportable read count of 180,000 (Figures 5A,C). There is a clone in this case with non-productive rearrangement with a stop codon present at nucleotide 109 position. The clone constitutes 70.7% of total reads, having V1-69 and J6 usage. Manual cross-check was performed using both IMGT/HighV-QUEST and IgBLAST programs to assess for any productive rearrangements and the same findings were confirmed (Figure 5B). The final interpretation is that the somatic mutation status can’t be determined due to the non-productive status of the clonal rearrangement. On flow cytometry, there was an absence of surface light chain expression (Figures 5D,E) supporting the non-productive nature of IGHV rearrangement.

### Case 3: Clonal Rearrangement Not Detectable by Leader or FR1 Primers

**FIGURE 6:**
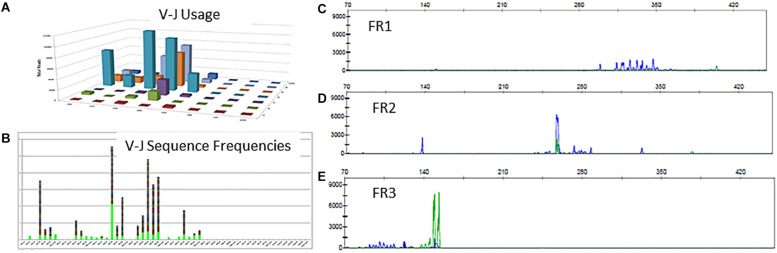
Clonal IGH sequences not detectable by leader, FR1 or FR3 sequences. Leader sequence-based NGS analysis shows no distinct clonal rearrangement **(A,B)**. Conventional PCR-based clonality assessment with capillary electrophoresis shows absence of clonal rearrangement by FR1 or FR3 primer sets (**C,E** - green tracing in E represents the FR3 positive control). The FR2 primer set shows a prominent clonal rearrangement **(D)**.

There is a polyclonal pattern with several dominant clones in the visual display of the Lymphotrack data output (Figures 6A,B). The analysis of IGH clonality assay revealed that the pattern is clonal with only FR2 primers (Figure 6D) and polyclonal with FR1 and FR3 primers (Figures 6C,E), indicating that the leader, FR1 and FR3 primers are failing here and only FR2 primers are detecting clonal rearrangements. So, the final evaluation is that the somatic mutation status can’t be determined due to sub-optimal amplification by leader primers. This additional analysis and documentation are consistent with the ERIC recommendations to perform analysis using an independent primer set when the primary primer set fails to detect a clonal rearrangement.

## Future Directions

Development of consensus validation guidelines, practice standards and specific quality assurance measures will be required to support increasing adoption of NGS-based SHM testing in clinical laboratories. The NGS analysis will likely result in detection of higher proportion of patients with multiple productive IGHV subclones, as seen by Stamatopoulos B. et al. (2017). This is expected due to the higher sensitivity of NGS. Also, compared with Sanger sequencing, NGS has better ability to amplify and reliably sequence mixtures of DNA templates. However, the detection of multiple clones and subclones with more sensitive NGS techniques challenges the conventional concept of “monoclonality” in lymphoid neoplasia, implying a new challenge to define the clinical significance of these additional data and findings. Hopefully, these new aspects of data interpretation can augment clinical utility in the areas of measurable residual disease detection, B-cell IG receptor stereotypy analysis and understanding disease transformation in CLL patients.

## Author Contributions

KP, SG, and DV contributed conception and design of the study. SG organized the reference database and wrote the first draft of the manuscript. KP and DV wrote sections of the manuscript, edited the manuscript, and contributed the figures. All authors contributed to manuscript revision, read and approved the submitted version.

## Conflict of Interest

The authors declare that the research was conducted in the absence of any commercial or financial relationships that could be construed as a potential conflict of interest.

## Footnotes

1 http://www.imgt.org/

2 https://www.ncbi.nlm.nih.gov/igblast/

3 https://bioinf-galaxian.erasmusmc.nl/argalaxy

4 www.ericll.org
